# Non-diabetic ketoacidosis secondary to primary hyperthyroidism: A case report

**DOI:** 10.1097/MD.0000000000028253

**Published:** 2022-06-10

**Authors:** Abdulrahman F. Al-Mashdali, Mohammadshah Gul, Waseem Umer, Abeer Omar, Akhnuwkh Jones

**Affiliations:** a Department of Internal Medicine, Hamad Medical Corporation, Doha, Qatar; b College of Medicine, Qatar University, Doha, Qatar.

**Keywords:** case report, hyperthyroidism, metabolic acidosis, non-diabetic ketoacidosis, thyrotoxicosis

## Abstract

**Introduction:**

There are variable complications of hyperthyroidism, including atrial fibrillation, heart failure, osteoporosis, and thyroid storm. One infrequent complication of hyperthyroidism is non-diabetic ketoacidosis (NDKA). To the best of our knowledge, our case is the third report of NDKA related to thyrotoxicosis.

**Patient concern:**

We describe a case of a 41-year-old African lady with no past medical history presented to our hospital with severe abdominal pain and vomiting for three weeks. This was associated with decreased appetite and weight loss.

**Diagnosis:**

Laboratory findings were significant for high anion gap metabolic acidosis, positive ketones in the urine, and high serum B-hydroxybutyrate. The blood glucose readings and HbA1c were within normal limits. Also, serum lactic acid and salicylate levels were within the normal range. The diagnosis of NDKA was made. Later, the thyroid functions test (TFT) confirmed the diagnosis of primary hyperthyroidism.

**Intervention and outcomes:**

The patient was managed initially with intravenous fluid and antiemetics. Then, she was started on propranolol and carbimazole. After which, her symptoms improved dramatically, and the metabolic acidosis (with serum ketones) were corrected within a few days of starting anti-thyroid medications.

**Conclusion:**

Despite its rarity, NDKA can be associated with severe thyrotoxicosis. Vigorous intravenous hydration and anti-thyroid medication are the mainstay treatment. TFT should be requested in a patient with unexplained NDKA

## 1 Introduction

Hyperthyroidism is a hormonal disorder with an excess of thyroid hormones (TH); thyroxine (T4) and triiodothyronine (T3).^[1]^ Symptoms include anxiety, emotional lability, weakness, tremor, palpitations, heat intolerance, increased perspiration, and weight loss despite a normal or increased appetite. Primary hyperthyroidism is diagnosed with low serum thyroid-stimulating hormone and high free T4 and/or T3 concentrations.^[2]^ The common complications of hyperthyroidism are heart failure, systolic hypertension, atrial fibrillation, osteoporosis weight loss primarily due to increased metabolic rate, and life-threatening condition known as thyrotoxicosis.^[3]^ Despite its extreme rarity, non-diabetic ketoacidosis (NDKA) might complicate severe hyperthyroidism.^[4]^ Herein; we describe a case of undiagnosed thyrotoxicosis presented initially with severe abdominal pain, nausea, vomiting, and ketoacidosis. Up to the best of our knowledge, this is the third reported case worldwide. This case report aims to shed light on this rare condition and outline our proposed management of this case.

## 2 Case presentation

A 41-year-old African lady with no past medical or surgical history presented to our emergency department with a three-week history of abdominal pain. The pain was progressive and localized mainly to the lower quadrants of the abdomen, cramping in nature, non-radiating, and 7 (out of 10) in severity. There were no aggravating or relieving factors. It was associated with nausea and frequent vomiting up to five times a day. She also had a loss of appetite for 3 weeks with about 3-kg loss. There was no previous history of similar complaints. She denied fever, shortness of breath, chest pain, cough, hemoptysis or hematemesis, dizziness, vaginal bleeding or discharge, dysuria, change in urine color, or increase in frequency. She denied the use of regular medications or herbal supplements. Her family history was significant for diabetes mellitus (DM) and hypertension (HTN). She denied smoking cigarettes, drinking alcohol, or using illicit drugs. Her menstrual cycle was regular, average amount, and lasting for 3 to 5 days.

Her vital signs were as follows: normal body temperature, tachycardia (110–115 beats/minute), blood pressure of 110/55 mm Hg, respiratory rate of 12 breaths/minute, normal oxygen saturation on room air. On physical examination, the abdomen was soft but with diffuse mild tenderness. There was no eyes manifestation of thyrotoxicosis, no goiter, and no fine tremor. However, there were features of proximal myopathy of the limbs, which was attributed initially to her poor nutritional status. Otherwise, examination of other systems was unremarkable. Initial investigation showed normocytic anemia, and blood chemistry was significant for high anion gap metabolic acidosis(HAGMA). Her kidneys function was within normal range. Urine analysis showed 4+ ketones with a high beta-hydroxybutyrate level (Table 1). Her Hba1c was 5.1%, and the readings of blood glucose were within the normal limit. Lactic acid and salicylate levels were within the normal range. She received continuous intravenous fluid (normal saline, alternating with dextrose + half normal saline) based on her body weight (around 60 mL/hour). Thyroid functions test revealed Thyroid-stimulating hormone < 0.01 mIU/L (reference range: 0.3–4.2), free T4 44.8 pmol/L (reference range: 11–23), and free T3 21.6 pmol/L (reference range: 3.7–6.4). Endocrinology service was consulted, and they started her on propranolol (80 mg daily) and carbimazole (20 mg daily). Anti-thyroid peroxidase and Anti-thyroid-stimulating hormone receptor were positive. Accordingly, she was diagnosed with Grave's disease.

**Table 1:** Relevant laboratory results during the hospital stay.

Parameter	Reference range	Day 1	Day 3	Day 6 (after starting carbimazole)	Day 8	Day 13 (discharge day)
pH	7.35-7.45	7.36	7.23	7.42	7.40	7.42
PCO2	35-45 mm Hg	34	26	32	38	37
Lactate	< 2	1.10	0.8	0.9	–	–
Bicarbonate	22-28 mm Hg	11	13	21.7	22	24
Sodium	135-145 mmol/L	138	138	140	139	137
Chloride	95–110 mmol/L	111	111	113	108	107
Potassium	3.5–5 mmol/L	3.7	3.9	4.1	4.3	3.8
Anion gap	6–12	16	14	5	9	6
B-hydroxybutyrate	<0.3 mmol/L	–	3.72	1.06	0.41	0.20

After starting the treatment, her abdominal pain, nausea, and vomiting improved within the first 72 hours. Also, her tachycardia and ketoacidosis resolved. On day 13 of the hospital stay, the patient was discharged home on propranolol and carbimazole. She was given a referral to endocrine clinic, but she lost follow-up since the date of discharge.

## 3 Discussion

Thyrotoxicosis is the clinical manifestation of a group of disorders characterized by excess TH action at the tissue level due to elevated TH concentration in the blood. Hyperthyroidism, a subset of thyrotoxicosis, refers to excess thyroid hormone synthesis and secretion by the thyroid gland. In the USA, the prevalence of hyperthyroidism is 1.2% (overt hyperthyroidism = 0.5%, subclinical hyperthyroidism = 0.7%). Graves’ disease is the most prevalent cause of hyperthyroidism in iodine-replete areas. It occurs more often in women, with the peak incidence in patients aged 30 to 60 years.^[5]^

Ketoacidosis is a metabolic state associated with pathologically high serum and urine concentrations of ketone bodies. Ketoacidoses broadly categorized into diabetic ketoacidosis, and NDKA. NDKA is defined as ketoacidosis in the absence of diabetes. The outcomes of NDKA cases are better than diabetic ketoacidosis cases, with almost complete recovery of all cases. The causes of NDKA include starvation, alcoholism, pregnancy, lactation, and a low carbohydrate diet. One of the rarest causes of NDKA is thyrotoxicosis.^[6]^

TH increase lipolysis by different mechanisms. Adipocytes in hyperthyroid patients were found to have increased numbers of beta- 2 adrenergic receptors and increased lipolytic response to beta-agonists. A recent showed that patients with thyrotoxicosis have elevated norepinephrine in subcutaneous adipose tissue compared to others, which might stimulate lipolysis by increasing the local release of NE. In addition, the carnitine shuttle transports long-chain fatty acids into the mitochondria of liver cells for beta-oxidation. TH might also act on this pathway to increase ketogenesis. So, the mechanisms mentioned above can support the effect of TH on lipolysis and the release of fatty acids from adipocytes that subsequently promote ketoacidosis.^[7–9]^

Our patient was found to have NDKA for unexplained etiology. Initially, given the main complaints of abdominal pain and nausea, we did not suspect thyrotoxicosis as an explanation of her presentation. However, we found that our patient had persistent sinus tachycardia and proximal myopathy, so we requested a thyroid function test that showed overt hyperthyroidism. After starting carbimazole, our patient had significant improvement in her symptoms. After an extensive literature review, we only found one case of hyperthyroidism with NDKA reported by Emily T. Wood and William B. Kinlaw in adults,^[4]^ and one case (presented as a poster) by Henry Jeng in children.^[10]^ In both cases, NDKA was the initial presentation of thyrotoxicosis, indicating that elevated TH levels are the main trigger for ketoacidosis. Also, all three cases,including ours, responded dramatically to anti-thyroid medication with hydration.

In conclusion, we should always have a high suspicion of hyperthyroidism in a patient presenting NDKA, especially in the typical population of middle-aged females in whom auto-immune conditions commonly encountered. Prompt initiation of anti-thyroid hormones medication is essential in the management of this condition.

## Acknowledgments

We would like to acknowledge the Qatar National Library (QNL) for open access publication funding of this article.

## Author contributions

**Conceptualization:** Abdulrahman F. Al-Mashdali, Mohammadshah Gul

**Data curation:** Abdulrahman F. Al-Mashdali, Mohammadshah Gul, Waseem Umer

**Writing – original draft:** Abdulrahman F. Al-Mashdali, Mohammadshah Gul, Waseem Umer, Abeer Omar

**Writing – review & editing:** Abdulrahman F. Al-Mashdali, Akhnuwkh Jones
